# Multicomponent carbohydrase system from *Trichoderma reesei*: A toolbox to address complexity of cell walls of plant substrates in animal feed

**DOI:** 10.1371/journal.pone.0251556

**Published:** 2021-06-04

**Authors:** Ninfa Rangel Pedersen, Morten Tovborg, Abdoreza Soleimani Farjam, Eduardo Antonio Della Pia

**Affiliations:** 1 Novozymes A/S Denmark, Kgs, Lyngby, Denmark; 2 Fermentationexperts A/S, Bække, Denmark; 3 Novozymes A/S Malaysia, Kuala Lumpur, Malaysia; INRAE, FRANCE

## Abstract

A diverse range of monocot and dicot grains and their by-products are commonly used in the animal feed industry. They all come with complex and variable cell wall structures which in turn contribute significant fiber to the complete feed. The cell wall is a highly interconnected matrix of various polysaccharides, proteins and lignin and, as such, requires a collaborative effort of different enzymes for its degradation. In this regard, we investigated the potential of a commercial multicomponent carbohydrase product from a wild type fermentation of *Trichoderma reesei* (*T*. *reesei*) (RONOZYME^®^ MultiGrain) in degrading cell wall components of wheat, barley, rye, de-oiled rice bran, sunflower, rapeseed and cassava. A total of thirty-one different enzyme proteins were identified in the *T*. *Reesei* carbohydrase product using liquid chromatography with tandem mass spectrometry LC-MS/MS including glycosyl hydrolases and carbohydrate esterases. As measured by *in vitro* incubations and non-starch polysaccharide component analysis, and visualization by immunocytochemistry and confocal microscopy imaging of immuno-labeled samples with confocal microscopy, the carbohydrase product effectively solubilized cellulolytic and hemicellulolytic polysaccharides present in the cell walls of all the feed ingredients evaluated. The *T*. *reesei* fermentation also decreased viscosity of arabinoxylan, xyloglucan, galactomannan and β-glucan substrates. Combination of several debranching enzymes including arabinofuranosidase, xylosidase, α-galactosidase, acetyl xylan esterase, and 4-O-methyl-glucuronoyl methylesterase with both GH10 and GH11 xylanases in the carbohydrase product resulted in effective hydrolyzation of heavily branched glucuronoarabinoxylans. The different β-glucanases (both endo-β-1,3(4)-glucanase and endo-β-1,3-glucanase), cellulases and a β-glucosidase in the *T*. *reesei* fermentation effectively reduced polymerization of both β-glucans and cellulose polysaccharides of viscous cereals grains (wheat, barley, rye and oat). Interestingly, the secretome of *T*. *reesei* contained significant amounts of an exceptional direct chain-cutting enzyme from the GH74 family (Cel74A, xyloglucan-specific β-1,4-endoglucanase), that strictly cleaves the xyloglucan backbone at the substituted regions. Here, we demonstrated that the balance of enzymes present in the *T*. *reesei* secretome is capable of degrading various cell wall components in both monocot and dicot plant raw material used as animal feed.

## Introduction

Modern feed industry is based on providing a precise composition of nutrients to meet specific requirements of production animals. However, in practice a degree of uncertainty remains between the formulated nutrients and the actual supplied nutrients. Such discrepancy is more prominent when it comes to non-starch polysaccharide composition. The specific structure and composition of non-starch polysaccharides in the grains not only vary by genotype, but also by the environment in which they are harvested. Plant cells dynamically remodel their cell wall structure and composition in attempt to adapt to the imposed environmental conditions [1]. Wheat grain grown under heat stress condition contain higher levels of arabinoxylan [2], and rice seedlings grown under cold stress condition contain higher levels of xyloglucan with more cross-linking to cellulose microfibers [3, 4]. A large-scale global survey of corn, wheat and barley quality indicated 14.39, 11.23 and 15.94% variation in crude fiber content of these grains, respectively [5]. The variation may be further enhanced if the measurement is narrowed down to specific polysaccharides. The arabinoxylan (A+X) content of wheat, barley, corn, rapeseed meal and soybean meal is reported to vary by 31, 32, 50, 16 and 41%, respectively [6]. This inherent variation brings high unpredictability to the end users of these grains and their by-products. This problem becomes crucial when considering that the size of the global feed production lies at over a billion tons annually and is expected to grow 1.6 times by 2050 [7]. Traditionally, the feed industry reduces the risks associated to inherent variation of grains nutrient composition with application of exogenous cell wall degrading enzymes. Enzymatic preparations with the highest enzyme activities are therefore regarded as the best tools to ensure the consistency of nutrient supply [8]. However, learnings from nature itself point out a different way whereby cell wall polysaccharides are degraded by adopting a right set and balance of enzymatic activities. The filamentous fungus *Trichoderma reesei* (*T*. *reesei*) is equipped with a unique secretome that acts in synergy to degrade polysaccharides present in cell walls of both monocot and dicot grains. This includes an array of enzyme activities to hydrolyze arabinoxylan, highly branched arabinoxylan (glucuronoxylan and arabino-glucuronoxylan), β-glucan, cellulose, xyloglucan, and pectin [9, 10]. The current study was designed to characterize the secretome of a wild type fermentation from *T*. *reesei* and its specific enzyme activities on various feed ingredients. We have previously demonstrated the efficacy of the same carbohydrase product to hydrolyze hemicellulose components of corn substrate with inherent variations [11, 12]. Here we expand the investigation to demonstrate the activity of the *T*. *reesei* fermentation product on multiple monocot and dicot grains such as wheat, barley, rye, oat, rice bran, rapeseed, sunflower and cassava. The effects of the multicomponent carbohydrase product are also demonstrated by confocal microscopy visualization that allows identification of individual polysaccharides within the intact cell wall structure, by-passing the shortcomings imposed by conventional purification and hydrolysis methods.

## Material and methods

### Enzymes

A multicomponent carbohydrase product obtained from a fermentation of *T*. *reesei* (RONOZYME^®^ MultiGrain (MultiGrain), DSM Nutritional Products, Basel, Switzerland) was used in this study. The product has endo-1,4-β-glucanase (EC 3.2.1.4; 800 U/g), endo-1,3(4)-β-glucanase (EC 3.2.1.6; 700 U/g), and endo-1,4-β-xylanase (EC 3.2.1.8; 2,700 U/g) as declared activities [13] and is active within a broad pH range (4–8). The recommended dosage of MultiGrain for poultry for fattening is 100 mg product per kg feed. Amyloglucosidase (AMG) (Megazyme, Bray, Ireland) and thermostable alpha-amylase (Termamyl 300 L, Novozymes, Denmark) were used for the double destarching step of the different plant materials.

### Plant material and chemicals

Whole grain wheat, barley and rye were obtained from Denmark. Oat, sunflower and rapeseed were obtained from France, de-oiled rice bran (DORB) and cassava from Thailand. All grain materials were milled to pass through a 0.5 mm sieve (ZM 200, Retsch, Haan, Germany). Rye arabinoxylan (RAX), wheat arabinoxylan (WAX), mixed linked (1–3),(1–4)-ß-D-glucan (MLG), β-galactomannan and xyloglucan were purchased from Megazyme, Ireland. All other chemicals were obtained from Sigma Aldrich (St. Louis, MO, U.S.A.).

### Protein identification by LC-MS/MS

Tryptic digests were prepared by a filter-aided sample preparation (FASP) method. Following digestion, the extracted peptides were analysed on a nano LC-MS/MS system: UltiMate 3000 RSLCnano / LTQ Orbitrap Velos Pro (Thermo/Dionex). For protein identification the data were searched against a specific database using the Mascot search engine (Matrix science) with Genedata Expressionist software having a 1% false discovery rate cut-off. Relative protein concentrations were calculated by label free quantification from peptide volumes using a Hi3 standard method in Genedata Expressionist.

### Viscosity measurements

Solutions of RAX (1 g/100 mL), WAX (1 g/100 mL), MLG (0.5 g/100 mL), β-galactomannan (1 g/100 mL), and xyloglucan (1 g/100 mL) were prepared in 0.1 M sodium acetate buffer, pH 6 to be used as substrates. Each substrate (250 μL) was pipetted to a 96 well microtiter plate (MTP) and incubated with 20 μL of the carbohydrase product, or buffer (control), at 40°C for 30 mins with stirring. The final carbohydrase product was diluted to a final concentration corresponding to 9 μg product per mL solution. Viscosity measurements were conducted using a Hamilton STARLET liquid handler (Hamilton) programmed to measure flow resistance pressure (Pa) during aspiration and dispensation as described in previous studies [14, 15].

### Double destarching of substrates

Destarching was carried out as described previously [16], albeit in a much smaller scale. In short, the five milled substrates wheat, barley, rye, oat and cassava were incubated with 2% alpha-amylase (Termamyl 300 L) at 90°C for 1 hour. The reaction mixture was cooled to 40°C, 14 U/mL of AMG was then added, and the solution was incubated in a shaking water bath overnight (16 hours). The procedure was repeated and then the destarched solids were collected by centrifugation and the pellets were washed 3 times with sodium acetate buffer and freeze dried. Analysis of the resulting material indicated that more than 99% of the starch present in the grains was removed by the double destarching process.

### *In vitro* incubations and monosaccharide analysis

To remove soluble oligomers and proteins, the milled rapeseed and sunflower samples were incubated in acetate buffer at pH 6.0 for 2 hours at 40°C. The solids were removed by centrifugation for 15 min at 4415 g and 5°C and freeze dried. The resulting rapeseed and sunflower samples, the double destarched wheat, rye, barley oat and cassava, and rice bran were incubated (10% dry matter) with the carbohydrase product at the commercial recommended dosage in acetate buffer pH 6.0 for 2 hours at 40°C with stirring (300 rpm). Experiments were run in quadruplicates. Following incubation with the multicomponent carbohydrase product, the solids were removed by centrifugation for 15 min at 4415 g and 5°C, and 500 μL of the supernatant was subjected to acid hydrolysis (1.6 M HCl) for 1 hour at 99°C. The solution was then neutralized with 1.3 M NaOH and the resulting samples were analyzed for neutral monosaccharide contents by high performance anion exchange chromatography combined with pulsed amperometric detection (HPAEC-PAD). Separation was achieved on a CarboPac analytical PA-210 column (id 2 mm) and a CarboPac PA-210 guard column (Thermofisher), at a temperature of 40°C with 1 mM KOH isocratic eluent flow rate of 0.2 mL/min. Seven monosaccharide standards (fucose, arabinose, rhamnose, galactose, glucose, xylose, mannose) were analyzed.

### Embedding, sectioning and staining

The procedure was conducted essentially as described before [16]. Pieces of approximately 4 × 4 × 6 mm of whole rye, barley, wheat, oat, rapeseed, sunflower (containing seed coat, aleurone layer and endosperm), cassava and DORB were fixed in Karnovsky’s fixative and washed in 0.1 M cacodylate buffer pH 7.3 and in demineralized water. The samples were then dehydrated in a graded series of ethanol before infiltration into melted paraplast (paraffin) at 60°C using Histochoice clearing agent (Sigma-Aldrich). Paraffin-embedded samples were sectioned into 7–10 μm thick sections on a 2030 Biocut microtome (Reichert-Jung, AU). De-paraffination was performed with Histochoice and a graded series of ethanol. Samples were stained with Calcofluor White for β-glucan and cellulose visualisation, and with Pontamine Fast Scarlet 4B for cellulose and xyloglucan visualisation.

### Incubations for microscopy studies

7–10 μm thick sections of de-paraffinated whole grains and DORB were treated with MultiGrain. 100 mg of the commercial multicomponent carbohydrase product were diluted in 5 mL 0.1 M sodium acetate buffer pH 6 containing 280 mg/L CaCl_2_. 50 μL of the obtained solution was applied directly on the microscope slide of the de-paraffinated samples. The samples were incubated with the commercial multicomponent carbohydrase product for 3 hours at 25°C under slight stirring in darkness. Controls were treated with buffer without the carbohydrase product at the same conditions. After 3 hours of incubation the samples were washed gently but thoroughly with deionized water and dried at room temperature. One set of slides was used for autofluorescence analysis and others for immunolocalization.

### Immunocytochemistry

Immunocytochemistry was performed on the de-paraffinated samples sections as described before [16]. Grain and antibody combinations were selected based on fit between each grain polysaccharide and the antigens used to raise the antibodies according to the manufacturer’s recommendations as well as prior experience with successful fluorescence imaging (Table 1). Samples were blocked with a 5% skimmed milk solution in 1x PBS for 1 hour and washed in PBS buffer followed by incubation with appropriate rat (LM6, LM11, LM25, LM28) or mouse monoclonal antibodies ((1,3),(1,4)-β-glucan antibody). Samples were subsequently incubated with a secondary antibody (anti-rat or anti-mouse IgG) linked to an Alexa-555 fluorophore and washed in PBS buffer. Citifluor AF1 anti-fading agent (Agar Scientific, UK) was added to avoid bleaching of fluorescence signal. Minimum two separate microscope slides were prepared for each specimen for both control and enzymatic treatment. A negative control was made by addition of only secondary antibody after blocking with 5% skimmed milk and washing with PBS buffer.

**Table 1:** Epitopes of commercial antibodies used in the study.

Antibody/ cat. no.	Epitope	Organism	Source
LM6	(1 → 5)-α-L-arabinan	Rat	PlantProbes (UK)
LM11	(1→4)-β-D-xylan/arabinoxylan	Rat	PlantProbes (UK)
LM25	Xyloglucan—binds to the XLLG, XXLG and XXXG oligosaccharides of xyloglucan	Rat	PlantProbes (UK)
LM28	Glucuronosyl residues in glucuronoarabinoxylan	Rat	PlantProbes (UK)
400–3	(1,3),(1,4)-β-glucan	Mouse	Biosupplies Australia Pty Ltd (Australia)

### Confocal laser scanning microscopy imaging

The confocal microscope IX83 Confocal Laser Scanning Microscope (Olympus, Japan) equipped with the software FV31S-SW software (Olympus, Japan) was used for confocal imaging. The following excitation and emission wavelengths were used: Alexa-555 and Pontamine Fast Scarlet (ex 561 nm/em 570–620 nm), Calcofluor White and autofluorescence (ex 405 nm/em 430–470 nm). Laser intensity for Calcofluor White images was 0.03% (in contrast to 3% for autofluorescence) which ensures neglectable detection of autofluorescence signal. Optimal image settings were selected prior to the final images acquisition. Images were then adjusted for brightness and contrast using same settings for images that had to be compared.

### Statistical analysis

Data analysis was performed using the software SAS JMP 15.1.0 (SAS Institute Inc., USA). Statistically significant differences were determined applying the Tukey-Kramer test at α = 0.05, as provided in the ANOVA method in the SAS JMP statistical package.

## Results

### Carbohydrase product composition

**Fig 1:**
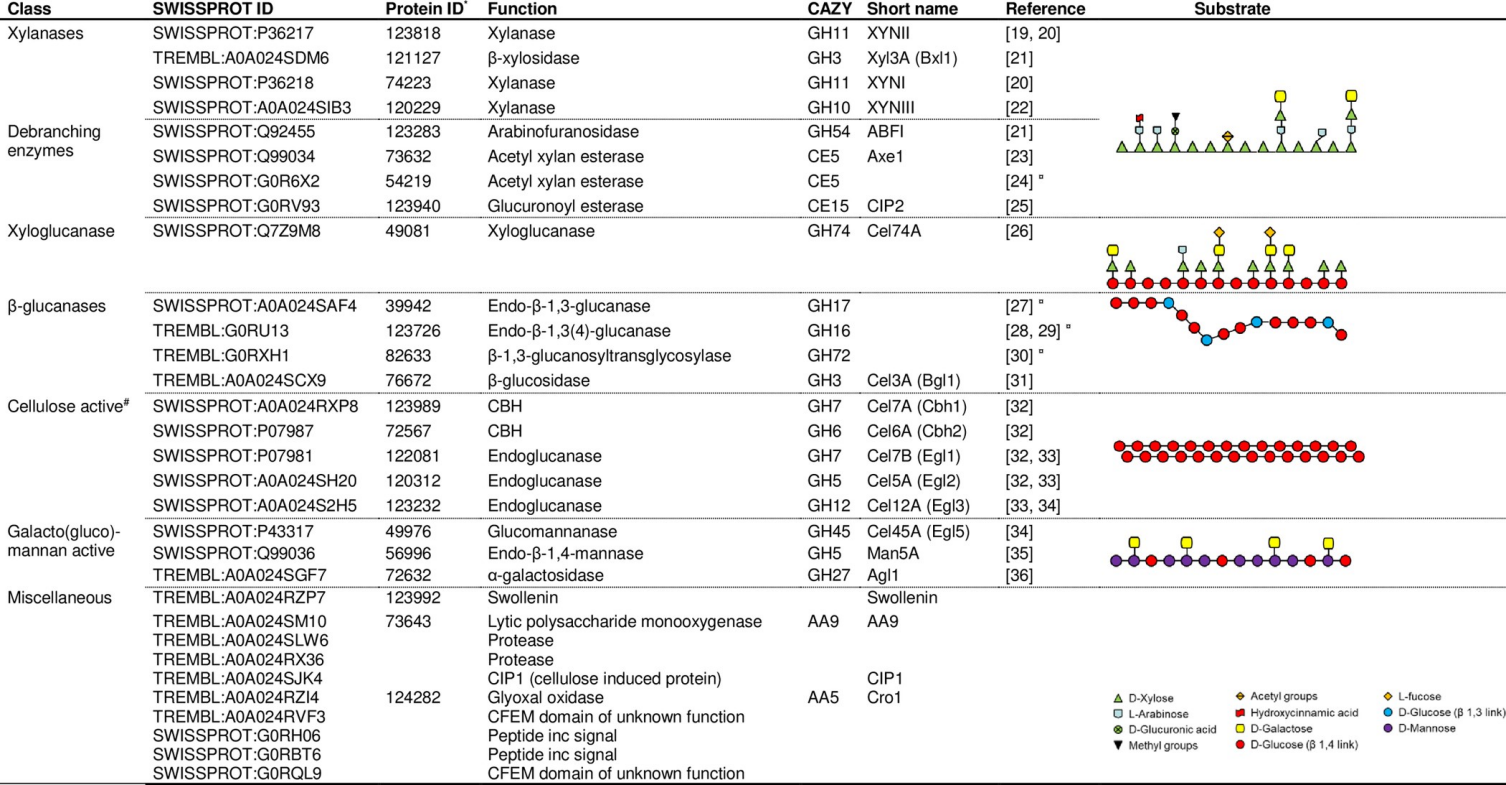
Proteins identified in MultiGrain grouped by enzyme class and substrate. References to characterization of the identical *T*. *reesei* enzyme are reported if available, otherwise reference to close fungal homologs are added to the table [19–36]. Structure diagrams are included to illustrate simplified polysaccharides commonly found in the discussed plant materials. * ID according to *T*. *reesei* database v2.0 [17], ^#^ Including endoglucanase, cellobiohydrolase, and betaglucosidase, ^¤^ Homolog characterized enzyme from different source.

Protein identification was performed on four independent production batches of MultiGrain and peptides were matched with the translated genome of *T*. *reesei*. The identified proteins are listed in Fig 1 with protein ID from v2.0 of the *T*. *reesei* database [17]. All identified carbohydrate active enzymes had identical hits in the database. More than thirty unique proteins were consistently found of which the majority were glycoside hydrolases with confirmed or predicted activity towards cell wall polysaccharides. In addition, several esterases with predicted debranching activity towards methyl and acetyl groups on hemicellulose were observed. Besides hydrolases and esterases there was a significant amount of non-hydrolytic proteins which also facilitates cell wall degradation. The most abundant ones were swollenin (ID 123992) and AA9 lytic polysaccharide monooxygenase (ID 73643). A low abundance of Chi46, a GH18 endo-N-acetyl-β-D-glucosaminidase, was also consistently found. A role of these enzymes in the modification of protein glycosylation (e.g. deglycosylation at the chitobiose core of N-glycans) has been suggested [18].

### Viscosity reduction

Within 30 minutes incubation of rye and wheat arabinoxylan, β-glucan, xyloglucan and galactomannan solutions, a notable and statistically significant viscosity reduction was observed in the multicomponent carbohydrase product-treated samples compared to the control samples (Table 2). These results indicated a clear degradation of viscous arabinoxylan, β-glucan, xyloglucan and galactomannan polymers. The viscosity reduction was substantial for rye and wheat arabinoxylan, β-glucan and xyloglucans, ranging from approximately 3x to 80x the reduction observed in the control samples. A lower, although still significant (P<0.05), viscosity reduction was observed for the solution containing galactomannan polymers (~2x reduction compared to the control sample).

**Table 2:** Viscosity reduction of solutions of rye- and wheat-arabinoxylan, β-glucan, xyloglucan and galactomannan upon treatment with MultiGrain^(^^a^^)^.

Treatment	Rye arabinoxylan ^(^^b^^)^	Wheat arabinoxylan	β-glucan	Xyloglucan	Galactomannan
Control	0.2 ± 0.5	3.0 ± 0.2	2.7 ± 1.4	5.3 ± 0.7	3.8 ± 0.2
MultiGrain	17.9 ± 1.1	17.1 ± 1.4	28.0 ± 1.3	13.4 ± 0.6	6.0 ± 0.5

(a) Viscosity measured as percentage change in flow resistance after 30 minutes incubation at 40°C supplemented with a control solution (deionized water) and MultiGrain.

(b) Results given as an average and standard error of determinations made in quadruplicate.

### Polysaccharide solubilization after *in vitro* incubation

The efficiency by which the multicomponent carbohydrase product could solubilize the polysaccharides of different cell walls was studied by incubating the four double destarched cereal grains (wheat, rye, barley and oat), rice bran, double destarched cassava and the two dicot grains with the carbohydrase product at the recommended commercial dosage. A significant (P<0.05) increase in the level of soluble monomers and oligomers (measured as glucose, arabinose, xylose and galactose in the supernatant after acid hydrolysis) was observed for all grains incubated with the multicomponent carbohydrase product (Table 3). No statistically significant difference in fucose, rhamnose and mannose concentrations (data not shown) was measured when the carbohydrase product-treated cereal grains were compared to the control incubations.

**Table 3:** Arabinose, galactose, glucose and xylose measured in the acid hydrolyzed supernatant of solution of different substrates incubated with a buffer solution (control) and MultiGrain for 2 hours at 40°C^(^^a^^)^.

Substrate	Treatment	Arabinose^(^^b^^)^	Galactose	Glucose	Xylose
Wheat	Control	0 ± 0	1.76 ± 0.03	2.17 ± 0.30	0 ± 0
	MultiGrain	41.04 ± 0.93	2.21 ± 0.03	10.24 ± 0.56	42.54 ± 0.97
Barley	Control	0 ± 0	1.43 ± 0.05	3.59 ± 0.15	0 ± 0
	MultiGrain	14.83 ± 1.03	1.96 ± 0.21	14.79 ± 0.42	9.73 ± 0.94
Rye	Control	0 ± 0	1.28 ± 0.04	4.21 ± 0.11	0 ± 0
	MultiGrain	37.49 ± 0.89	1.81 ± 0.35	27.20 ± 0.90	38.74 ± 2.68
Oat	Control	0 ± 0	1.95 ± 0.11	1.57 ± 0.06	0 ± 0
	MultiGrain	31.08 ± 0.45	2.38 ± 0.09	9.84 ± 0.13	34.65 ± 0.32
Rice bran	Control	0.13 ± 0.01	0.13 ± 0.01	0.27 ± 0.03	0.06 ± 0.01
	MultiGrain	0.41 ± 0.01	0.20 ± 0.01	0.60 ± 0.03	0.51 ± 0.01
Rapeseed	Control	2.99 ± 0.2	2.43 ± 0.13	7.80 ± 0.16	0.40 ± 0.08
	MultiGrain	3.50 ± 0.2	2.81 ± 0.11	8.74 ± 0.2	1.26 ± 0.09
Sunflower	Control	1.22 ± 0.04	2.26 ± 0.13	5.04 ± 0.22	0.20 ± 0.05
	MultiGrain	1.47± 0.04	2.31 ± 0.08	5.22 ± 0.19	0.53 ± 0.06
Cassava	Control	0.16 ± 0.01	0.35 ± 0.01	0.65 ± 0.01	0.07 ± 0.01
	MultiGrain	0.26 ± 0.01	0.49 ± 0.01	0.92 ± 0.02	0.40 ± 0.01

^(a)^ Monosaccharides are given as mg per g of the total destarched dry matter.

^(b)^ Results given as an average and standard error of determinations made in quadruplicate.

### Confocal microscopy imaging

#### Cereals

**Fig 2:**
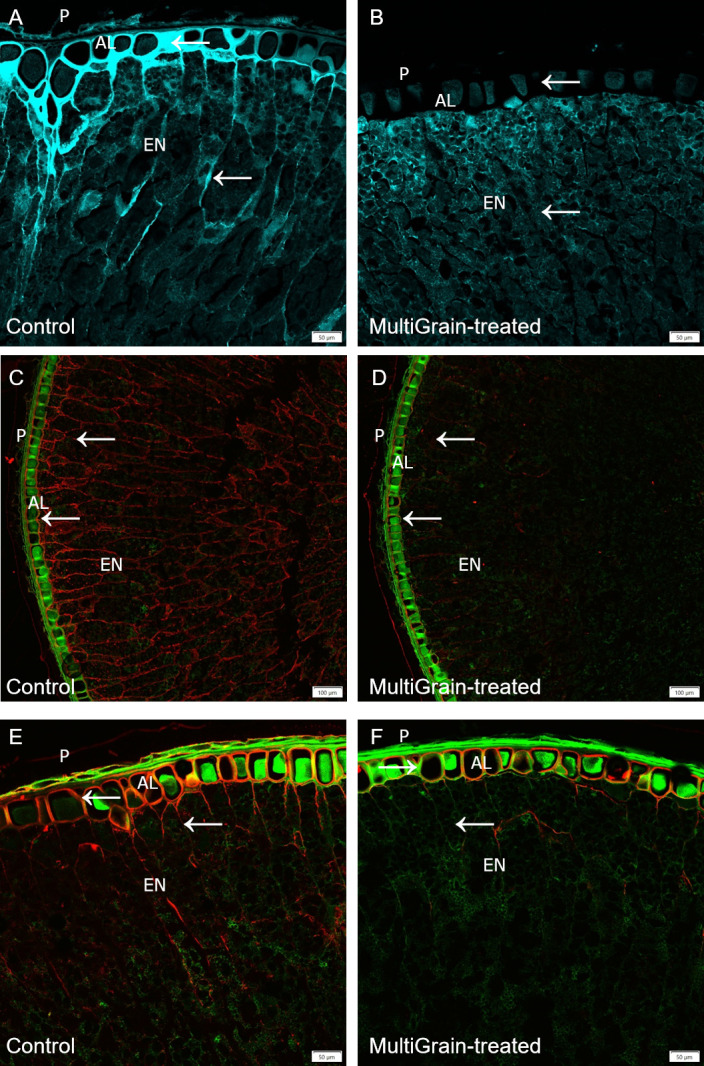
Confocal images of cross sections of whole wheat grains. Section of samples incubated (A, C, E) with sodium acetate buffer (control) or (B, D, F) with MultiGrain for 3 hours at pH 5. The sections were subsequently (A, B) stained with Calcoflour White, or (C, D) incubated with the LM11 antibody detecting arabinoxylans with low degree of branching and the red-fluorescence labelled secondary antibody, or (E, F) incubated with the antibody detecting (1,3),(1,4)-β-glucan and the red-fluorescence labelled secondary antibody. (A, B) Light blue signal indicates cellulose and other β-1,4-linked glucans. Red signal indicates (C, D) arabinoxylans and (E, F) β-glucans and green signal indicates autofluorescence. P = pericarp; AL = aleurone; EN = starchy endosperm. Arrows point to sites where the fluorescence signal due to staining or immunolabelling is present and removed following treatment with MultiGrain. (A, B) Scale bar = 50 μm, (C, D) Scale bar = 100 μm, (E, F) Scale bar = 50 μm.

The confocal microscopy images of wheat grain cross sections are illustrated in Fig 2. Fig 2A shows a wheat sample stained with calcofluor white which selectively binds cellulose and other β-1, 4-linked glucans [37]. The cell walls of the aleurone layer were stained more intense than those in the endosperm. Following incubation with the carbohydrase product, both signals almost disappeared (Fig 2B). High presence of arabinoxylan (labelled with LM11 antibody) was detected in the cell walls of the endosperm (Fig 2C), and β-glucans in the aleurone layer (labelled with antibody against (1,3),(1,4)-β-glucans) (Fig 2E). Treatment with MultiGrain removed almost completely the epitopes recognized by both antibodies and only a low fluorescence signal was detected at the interface between the aleurone and sub-aleurone layer (Fig 2D and 2F). No fluorescence signal was detected when slides were immunolabelled with the LM28 antibody detecting branched glucuronoarabinoxylans (S1 Fig).

**Fig 3:**
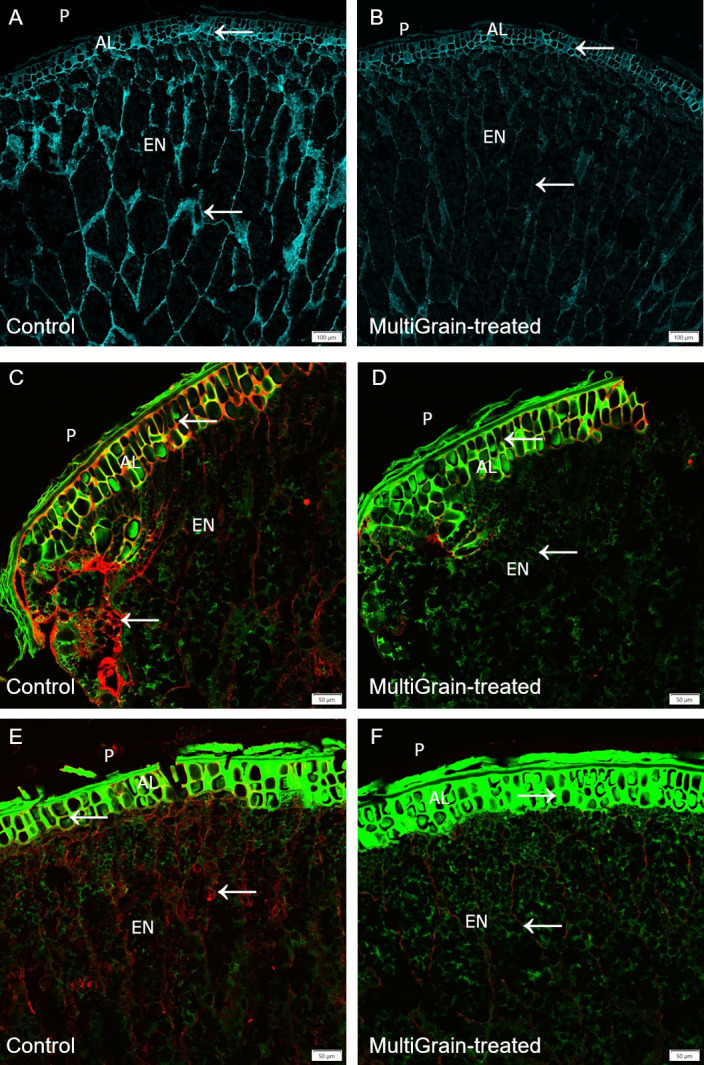
Confocal images of cross sections of whole barley grains. Section of samples incubated (A, C, E) with sodium acetate buffer (control) or (B, D, F) with MultiGrain for 3 hours at pH 5. The sections were subsequently (A, B) stained with Calcoflour White, or (C, D) incubated with the LM11 antibody detecting arabinoxylans with low degree of branching and the red-fluorescence labelled secondary antibody, or (E, F) incubated with the antibody detecting (1,3),(1,4)-β-glucan and the red-fluorescence labelled secondary antibody. (A, B) Light blue signal indicates cellulose and other β-1,4-linked glucans. Red signal indicates (C, D) arabinoxylans, (E, F) β-glucans and the green signal indicates autofluorescence. P = pericarp; AL = aleurone; EN = starchy endosperm. Arrows point to sites where the fluorescence signal due to staining or immunolabelling is present and removed following treatment with MultiGrain. (A, B) Scale bar = 100 μm. (C-F) Scale bar = 50 μm.

Cell wall structures of the pericarp, aleurone, subaleurone layers and the starchy endosperm of the barley samples were clearly detectable by Calcofluor White staining (Fig 3A). MultiGrain treatment mostly removed the fibers in the endosperm and subaleurone layer (Fig 3B). The confocal images of barley immunolabelled with the LM28 antibody did not reveal any fluorescence signal (S2 Fig), while the ones immunolabelled with the LM11 antibody indicated variable amount of arabinoxylans (Fig 3C) [16]. Following incubation with the carbohydrase product, the arabinoxylan structures almost disappeared (Fig 3D). Abundant presence of β-glucans was detected in the cell walls of the endosperm (Fig 3E). These structures disappeared completely upon incubation with the carbohydrase product (Fig 3F).

**Fig 4:**
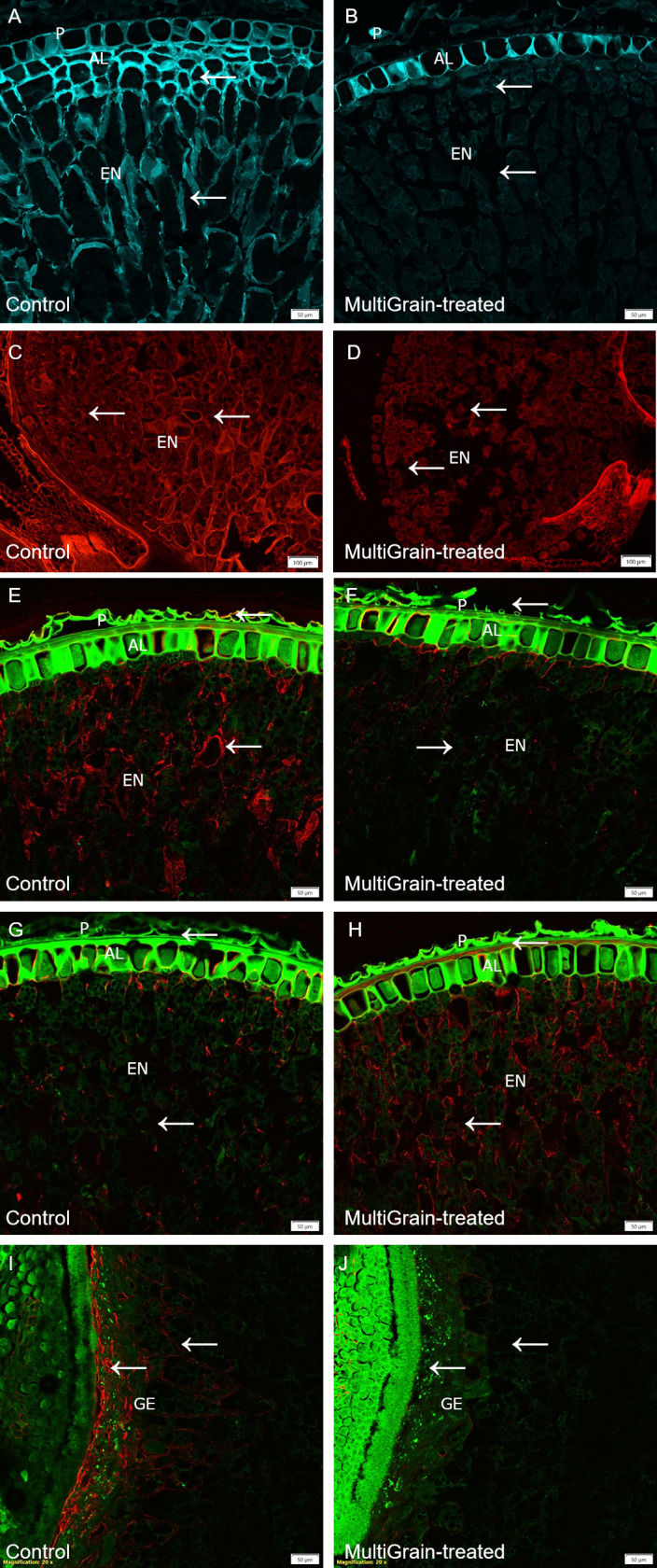
Confocal images of cross sections of whole rye grains. Section of samples incubated (A, C, E, G, I) with sodium acetate buffer (control) or (B, D, F, H, J) with MultiGrain for 3 hours at pH 5. The sections were subsequently (A, B) stained with Calcoflour White, or (C, D) stained with Pontamine Fast Scarlet, or (E, F) incubated with the LM11 antibody detecting arabinoxylans with low degree of branching and the red-fluorescence labelled secondary antibody, or (G, H) incubated with the antibody detecting (1,3),(1,4)-β-glucan and the red-fluorescence labelled secondary antibody, or (I, J) incubated with the LM28 antibody detecting glucuronoarabinoxylan with high degree of branching and the red-fluorescence labelled secondary antibody. (A, B) Light blue signal indicates cellulose and other β-1,4-linked glucans. Red signal indicates (C, D) cellulose structures, (E, F) arabinoxylans, (G, H) β-glucans, (I, J) glucuronoarabinoxylans and the green signal indicates autofluorescence. P = pericarp; AL = aleurone; EN = starchy endosperm, GE = germ. Arrows point to sites where the fluorescence signal due to staining or immunolabelling is present and removed following treatment with MultiGrain. (A-D) Scale bar = 100 μm. (E-J) Scale bar = 50 μm.

Aleurone layer and endosperm cell walls of rye grains stained intensely with Calcofluor White (Fig 4A). Carbohydrase product treatment of the sections removed stainable material from the starchy endosperm cell walls completely (Fig 4B). Staining samples with Pontamine Fast Scarlet, a dye that fluoresces preferentially in the presence of cellulose and xyloglucan [38, 39], resulted in a strong fluorescence signal from the endosperm cell walls (Fig 4C). Upon incubation with the multicomponent carbohydrase product, the fluorescence signal completely disappeared (Fig 4D). While the LM11 and β-glucan antibodies bound to the cell walls of the starchy endosperm and aleurone layer (Fig 4E and 4G, respectively), the LM28 antibody bound only to the cell walls in the germ (Fig 4I). Following incubation with the carbohydrase product the fluorescence signal disappeared and only a low fluorescence signal was measured in the sub-aleurone layer or throughput the endosperm (Fig 4F, 4H and 4J).

**Fig 5:**
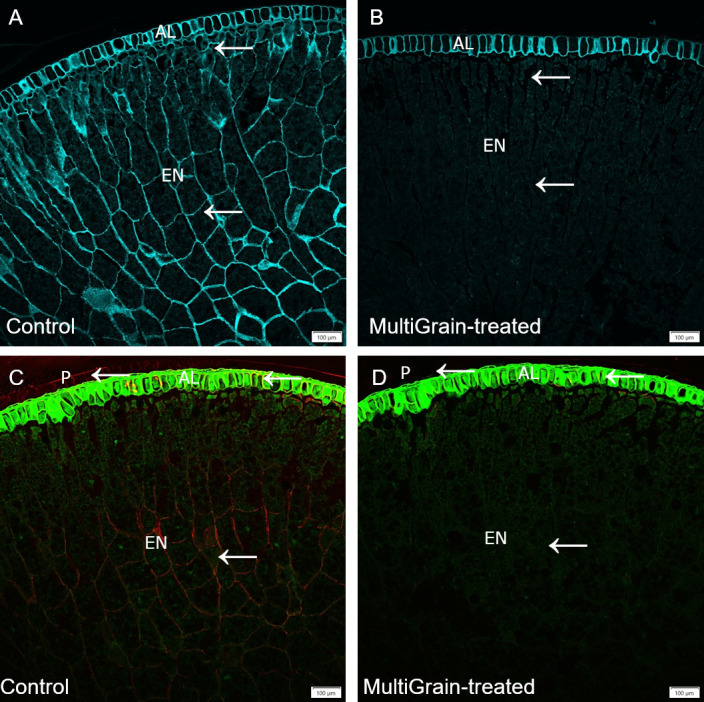
Confocal images of cross sections of whole oat grains. Section of samples incubated (A, C) with sodium acetate buffer (control) or (B, D) with MultiGrain for 3 hours at pH 5. The sections were subsequently (A, B) stained with Calcofluor White, or (C, D) incubated with the antibody detecting (1,3),(1,4)-β-glucan and the red-fluorescence labelled secondary antibody. (A, B) Light blue signal indicates cellulose and other β-1,4-linked glucans. Red signal indicates (C, D) β-glucans and the green signal indicates autofluorescence. AL = aleurone; EN = starchy endosperm. Arrows point to sites where the fluorescence signal due to staining or immunolabelling is present and removed following treatment with MultiGrain. (A, B) Scale bar = 100 μm. (C, D) Scale bar = 50 μm.

Both the cell walls of the endosperm and aleurone layer of the oat grain were clearly stained with Calcofluor White (Figs 5A and S3A). The fluorescence signal was removed completely in the endosperm and partly in the aleurone layer after treatment with the carbohydrase product (Figs 5B and S3B). β-glucans presence in oat was mostly evident in the pericarp and endosperm (Figs 5C and S4A). Treatment with the multicomponent carbohydrase product removed completely the epitopes recognized by the anti-β-glucan antibody (Figs 5D and S4B).

**Fig 6:**
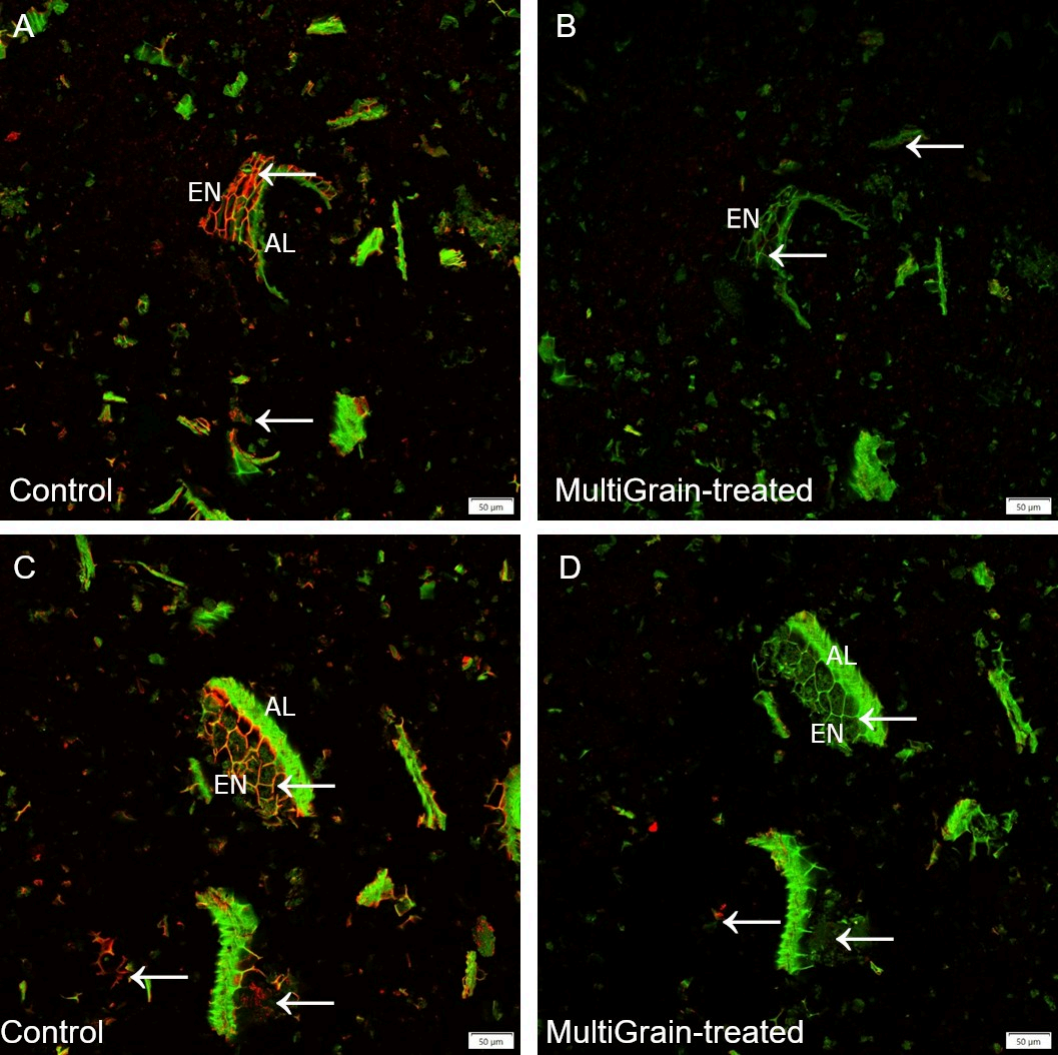
Confocal images of cross sections of DORB. Section of samples incubated (A, C) with sodium acetate buffer (control) or (B, D) with MultiGrain for 3 hours at pH 5. The sections were subsequently (A, B) incubated with the LM28 antibody detecting arabinoxylans and the red-fluorescence labelled secondary antibody or (C, D) incubated with the LM25 antibody detecting xyloglucans with different branching structures and the red-fluorescence labelled secondary antibody. Red signal indicates (A, B) glucuronoarabinoxylans and (C, D) xyloglucans and the green signal indicates autofluorescence. AL = aleurone; EN = starchy endosperm. Arrows point to sites where the fluorescence signal due to staining or immunolabelling is present and removed following treatment with MultiGrain. (A-D) Scale bar = 50 μm.

Industrial thermal and mechanical pretreatment of whole rice to obtain the DORB samples results in a very complex cell wall structure. The microscopy pictures reported in Fig 6 are representative of several analyzed samples. Imaging of the DORB samples showed that while processing destroyed most of the starchy endosperm cell wall structure, the rice pericarp and aleurone layer were resistant to the processing method (Fig 6A and 6C). The DORB samples showed high auto-fluorescence signal most likely due to the high content of ferulic acid in the outer cell wall structure (Fig 6A and 6C) [40]. Intensity of the autofluorescence signal decreased after carbohydrase product treatment (Fig 6B and 6D). Immunolabelling with LM28 and LM25 antibodies showed that complex glucuronoarabinoxylans and xyloglucans were present in the samples (Fig 6A and 6C, respectively). Neither of the fluorescence signals were detectable upon incubation with the carbohydrase product (Fig 6B and 6D). No arabinoxylans with low degree of branching were detected in the samples as visualized by incubation with LM11 antibody immunolabelling (S5 Fig).

#### Dicotyledons

**Fig 7:**
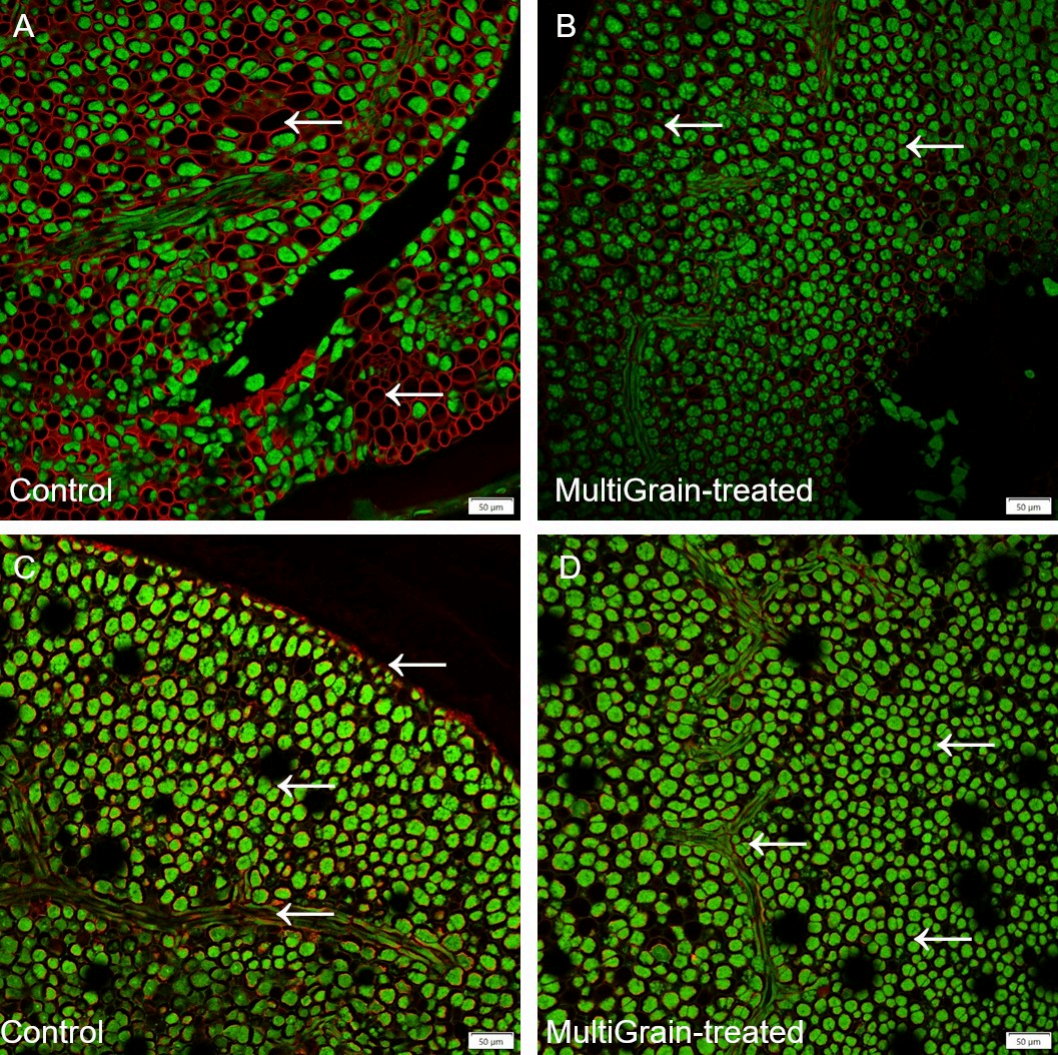
Confocal images of cross sections of whole rapeseed seeds. Sections of sample incubated (A, C) with sodium acetate buffer (control) or (B, D) with MultiGrain for 3 hours at pH 5. Slides were subsequently incubated with (A, B) the LM25 antibody detecting xyloglucans or (C, D) with the LM6 antibody detecting (1–5)-α-L-arabinans. Red fluorescence labelled secondary antibody used for visualization of sections. Red signal indicates (A, B) xyloglucans or (C, D) arabinans and the green signal indicates autofluorescence from proteins bodies. Arrows point to sites where the fluorescence signal due to staining or immunolabelling is present and removed following treatment with MultiGrain. Scale bar = 50 μm.

Confocal images of rapeseed sections immunolabelled with LM25 and LM6 antibodies are illustrated in Fig 7A and 7C, respectively. The immunolabelling fluorescence signal due to binding of the LM25 antibodies that recognize different substituted epitopes in highly branched xyloglucan was very intense and homogeneous in the cotyledon cell walls (Figs 7A and S6A). The fluorescence signal due to the immunofluorescent probe LM6 detecting (1–5)-a-L-arabinans however was mostly concentrated in the seed coat with minor presence in the cell walls of the cotyledon (Fig 7C). Upon incubation with the carbohydrase product the fluorescence signal notably reduced in intensity indicating removal of the epitopes recognized by both the LM25 and LM6 antibodies (Figs 7B and 7D and S6B and S7). The micrographs of rapeseed show autofluorescence from protein bodies within the cell walls upon UV-excitation (coloured in green in Fig 7A and 7C). Following incubation with MultiGrain, the autofluorescence signal of the protein bodies decreased in intensity and became less homogeneous (Fig 7B and 7D).

**Fig 8:**
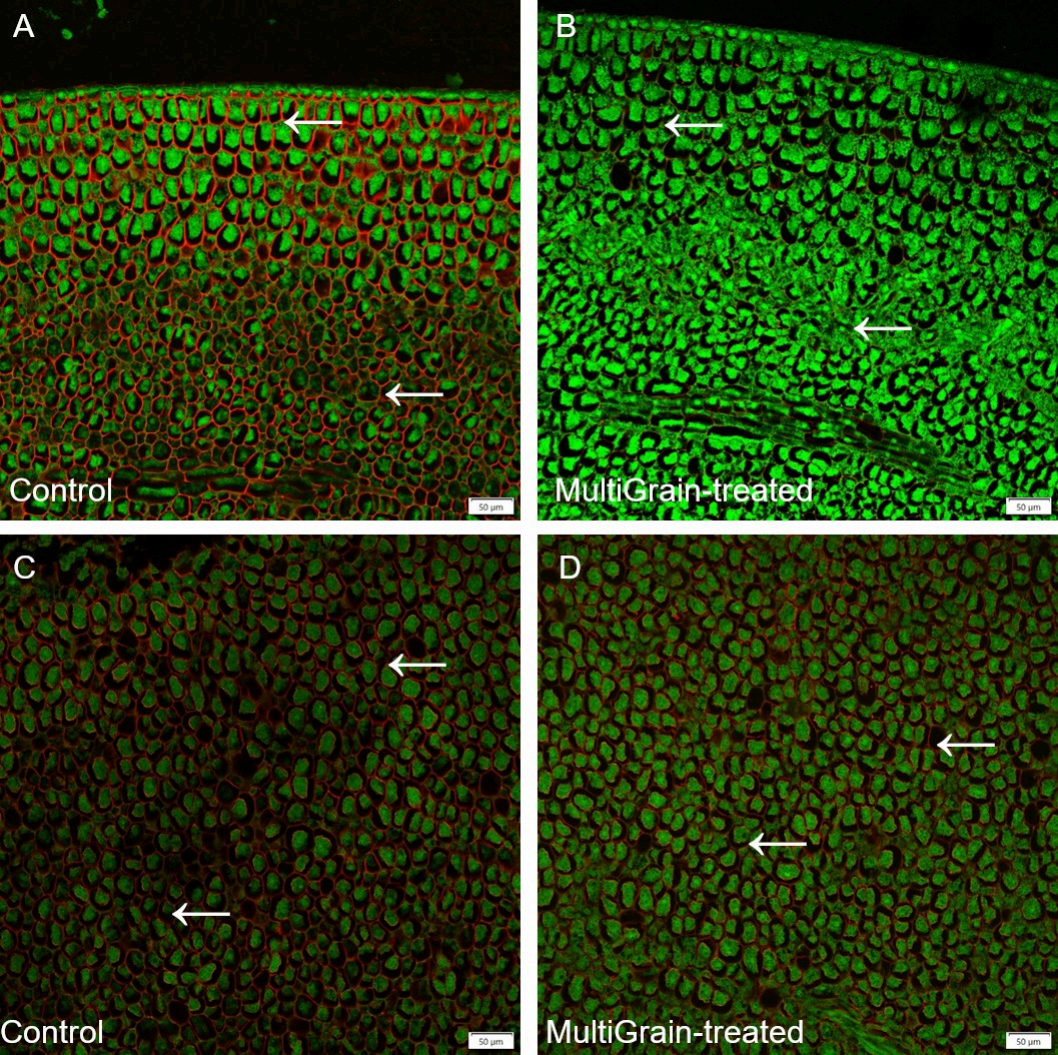
Confocal images of cross sections of whole sunflower seeds. Sections of sample incubated (A, C) with sodium acetate buffer (control) or (B, D) with MultiGrain for 3 hours at pH 5. Slides were subsequently incubated (A, B) with the LM25 antibody detecting xyloglucans with different branching structures or (C, D) with the antibody detecting (1–5)-α-L-arabinans. Red fluorescence labelled secondary antibody used for visualization of sections. Red signal indicates (A, B) xyloglucans having highly branched substitutions or (C, D) **(**1–5)-α-L-arabinans and the green signal indicates autofluorescence from proteins bodies. Arrows point to sites where the fluorescence signal due to staining or immunolabelling is present and removed following treatment with MultiGrain. Scale bar = 50 μm.

The presence of xyloglucans and (1–5)-α-L-arabinans in sunflower seed cross sections was visualised by immunolabelling with antibodies LM25 and LM6, respectively (Fig 8A and 8C). Both the antibodies bound homogeneously to the cell wall structures in the cotyledon (Fig 8A and 8C). After incubation with the carbohydrase product, the xyloglucans signal completely disappeared (Fig 8B), while a decrease in arabinan signal was observed (Fig 8D).

**Fig 9:**
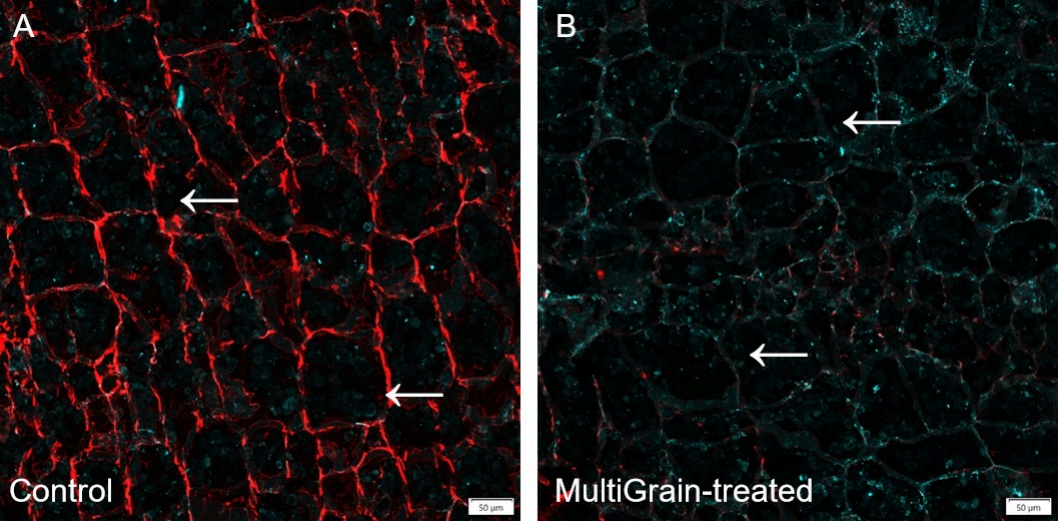
Confocal images of cross sections of whole cassava root. Sections of sample incubated with (A) sodium acetate buffer (control) or (B) with MultiGrain for 3 hours at pH 5. Slides were subsequently incubated with the LM25 antibody detecting xyloglucans and the red fluorescence labelled secondary antibody. Red signal indicates xyloglucans with different branching structures and the blue signal indicates autofluorescence from cell walls structures. Arrows point to sites where the fluorescence signal due to staining or immunolabelling is present and removed following treatment with MultiGrain. Scale bar = 50 μm.

The presence of xyloglucans in cassava root cross sections was visualised by immunolabelling with antibodies LM25 (Fig 9). The antibodies bound homogeneously to the cell wall structures in the tuberous root (Fig 9A). Following incubation with MultiGrain, the xyloglucans signal decreased in intensity (Fig 9B). No fluorescence signal was detected when slides were immunolabelled with the LM6 antibody detecting (1–5)-α-L-arabinans (S8 Fig).

## Discussion

Plant cell walls have complex structures that differ significantly across species and botanic structures. Furthermore, different cell types in the same species have unique cell walls that differ in content and composition of their polysaccharide components. The insoluble and tightly packed nature of the cell wall fibers severely limits access to nutrients such as starch and protein by the primary endogenous enzymes during gastrointestinal passage. *T*. *reesei* is a common filamentous aerobic fungus used for industrial applications [10]. It is well known for its great potential for enzyme production and its genome contains a wealth of genes coding for cell wall-degrading enzymes [10, 41]. Protein identification of MultiGrain, a specific commercial multicomponent carbohydrase preparation of *T*. *reesei*, revealed thirty-one unique proteins (Fig 1), many of which correspond to previous reports of *T*. *reesei* proteomics [10, 42, 43]. Most of the identified proteins are glycosyl hydrolases and carbohydrate esterases with predicted activity on cell wall polysaccharides.

A total of four xylanases were identified. The neutral pH optimum GH11 xylanase XYNII [19] was the dominating xylanase in the *T*. *Reesei* fermentation and it was complemented with the neutral GH10 xylanase XYNIII [22] and the more acidic GH11 xylanase XYNI [20]. Another xylanase XYNIV was shown to have both endo- and exo- activities on arabinoxylan and glucuronoarabinoxylan [44]. Two other enzymes belonging to the xylan-degrading enzymes group were also identified; a beta-xylosidase (Xyl3A) and an arabinoxylan-debranching enzyme arabinofuranosidase (ABFI), the latter of which cleaves off arabinofuranosyl substitutions improving the xylan backbone accessibility to GH10 and GH11 xylanases [21]. Three carbohydrate esterases were identified. CIP2 is a CE15 enzyme specific for hydrolyzing ester linkages between alcohol groups in lignin and the acid of methyl-glucuronyl group in glucuronoxylan [25, 45]. The CE15 glucuronoyl esterase is therefore responsible for detaching glucuronoxylan from lignin and hence making the polysaccharide more available for carbohydrases. One of the two CE5 acetyl xylan esterases (Axe1) has been confirmed to have acetyl xylan esterase activity [23]. Incubation of the *T*. *reesei* carbohydrase product with four cereal grains (wheat, barley, rye and oat) confirmed that the xylanases were active on different feed raw materials. In fact, neutral monosaccharide analysis of the acid hydrolyzed solubilized fraction of untreated and MultiGrain-treated samples indicated that arabinoxylans were hydrolyzed into soluble monomers and oligomers (Table 3). Interestingly, galactose was also identified in the neutral monosaccharide analysis of all the MultiGrain-treated samples. This could indicate that galactose was also present on the side chains of the solubilized arabinoxylan oligomers. The confocal microscopy pictures also demonstrated how MultiGrain could hydrolyze both low and highly-substituted arabinoxylans (Figs 2C, 2D, 3C, 3D, 4E, 4F, 6A and 6B). Hydrolysis of different arabinoxylan structures by the multicomponent carbohydrase was clearly confirmed by the immunolabelling of the cereals cell walls and subsequent confocal microscopy analysis. Presence of the beta-xylosidase, the arabinofuranosidase, the GH27 alpha-galactosidase and the three carbohydrate esterases in the carbohydrase product is fundamental to cleave the highly substituted arabinoxylans like the ones present in rice bran (Fig 6A and 6B) and rye (Fig 4I and 4J). In fact, these glycosyl hydrolases and esterases act synergistically with the xylanases by cleaving arabinose, xylose, galactose, acetyl groups and the other substituents from the xylan backbone and leaving it accessible to the GH10 and GH11 xylanases. The current study clearly demonstrated that the carbohydrase product from *T*. *reesei* could hydrolyze arabinoxylans with different levels of substitution into oligomers such as arabinoxylo-oligosaccharides (AXOS). Recent work in our laboratory showed that combination of a xylanase and an α-arabinofuranosidase can effectively solubilize complex glucuronoarabinoxylans into AXOS [46]. Oligomers such as AXOS and XOS are generally known to have prebiotic effects enhancing gut functionality, feed conversion ratio and body weight gain [45–47].

Soluble arabinoxylans are known to bind water leading to high viscosity of the intestinal content, reducing both nutrients digestion and feed intake capacity while increasing intestinal inflammation [47, 48]. Xylanase-based products are commonly used in the feed industry to reduce the antinutritional effects of arabinoxylans. It is widely recognized that these carbohydrases exert in part their beneficial effects by reducing the viscosity of the intestinal content [49, 50]. Viscosity measurements of extracted soluble arabinoxylans incubated in presence of MultiGrain (Table 2) clearly demonstrated that the xylanases of the multicomponent carbohydrase product were effective in depolymerizing soluble arabinoxylans from both wheat and rye.

Mixed linked (1→3), (1→4)-β-D-glucans (β-glucans) represent the second most abundant non-starch polysaccharides fraction in wheat, barley, rye and oat [51]. Three enzymes with specific activity on β-glucan were identified in MultiGrain (Fig 1). They belong to families GH16, GH17, and GH72, and have different mechanisms in their β-glucan hydrolysis. The GH17 and GH72 enzymes both hydrolyze internal β-1,3-linkages. In the hydrolysis process the GH17 introduces short terminal β-1,6 branches on the glucan [27] and the GH72 elongates another β-glucan on the non-reducing end [30]. The endo-β-1,3(4)-glucanase from GH16 was shown to degrade mixed linkage β-glucan from barley by specifically hydrolyzing internal β-1,3 linkage [28, 29]. There were also three endoglucanases (Cel5A, Cel7B, cel12A) and a β-glucosidase Cel3A in the *T*. *reesei* fermentation that have β-glucanase activity, in addition to activity on glucomannan which complement the glucomannanase Cel45A [34]. The immunofluorescence microscopy experiments confirmed that *T*. *reesei* carbohydrase product removed β-glucan epitopes [52] from both the aleurone and endosperm cell structures of wheat (Fig 2E and 2F), barley (Fig 3E and 3F), rye (Fig 4G and 4H) and oat (Fig 5C and 5D). A high degree of solubilization of β-glucans was also confirmed by the results of the *in vitro* incubations of the four destarched cereal grains (Table 3). The acid hydrolyzed solubilized fraction of the samples treated with MultiGrain showed five to six times more glucose than control samples. Barley and oat have high levels of viscous soluble β-glucans [50] that most likely cause decrease in animal performance. Several researchers have described the negative effects of soluble β-glucans in barley and demonstrated that the addition of β-glucanase to broilers diets can ameliorate the viscosity problems associated with barley [53]. Addition of MultiGrain to soluble β-glucans solutions clearly demonstrated that the different β-glucanases present in the *T*. *reesei* carbohydrase product effectively reduced the viscosity of the soluble pentosans medium by shortening soluble β-glucan chains (Table 2). Our observations support the findings of Brenes *et al*. that demonstrated that addition of MultiGrain to highly viscous barley-based diets significantly improves broilers performance parameters and reduces the size of the pancreas and of the gastrointestinal tract [54].

Several enzymes directly active in cellulose degradation were identified in the *T*. *reesei* carbohydrase product. These were the cellobiohydrolases CBH1 (Cel7A) and CBH2 (Cel6A), the endoglucanases (Cel7B, Cel5A, Cel12A), and the β-glucosidase (Cel3A). All of these play a role in degrading crystalline and amorphous cellulose [31, 32, 34]. Lytic polysaccharide monooxygenase (LPMO) (AA9) is also present. The LPMO from family AA9 has a key role in lignocellulose degradation. Even though the LPMO would be predicted to have little impact in the largely anaerobic intestinal environment, recent studies have shown that H_2_O_2_ can replace O_2_ for LPMOs to break the glycosidic bonds [55]. In this study we used Calcofluor White to stain cellulose in selected cereal grains as seen in Fig 2 (wheat), Fig 3 (barley), Fig 4 (rye), and Fig 5 (oat). Calcofluor White also stains other β-1,4-glucan structures such as cellulose and callose. In all the examples above we observed Calcofluor White signal both in endosperm and aleurone cell walls, but in general the signal is stronger in the aleurone layer. Although cellulose is more abundant in the outer seed coat, it is also present in the aleurone cell wall [37]. In all four cereals, incubation with the carbohydrase product significantly reduced the fluorescence signal. In the aleurone layer there was a strong reduction in fluorescence signal suggesting degradation of cellulose and β-glucan structures. In the endosperm, where the signal was most likely from β-glucan, the fluorescence signal was almost complete lost. The cell walls in the aleurone cells appeared to differ in resistance to degradation by the carbohydrase product most likely due to differences in β-glucan branched structure. In the wheat sample the signal was completely lost, whereas the aleurone cell walls were still visible in the remaining cereals. Part of the lignocellulosic pericarp in rye was visible on the outside of the aleurone layer stained with Pontamine Fast Scarlet (Fig 4C). In the sample treated with MultiGrain not only were the cell walls of the endosperm missing, but most of the pericarp was either degraded or detached and lost resulting in full exposure of the nutrient rich aleurone cells.

GH54 arabinofuranosidases are exo-1,5-acting and typically ascribed the function of removing α-1,5-linked arabinosyl branches in arabinoxylan as described above. However, they are not necessarily specific for this single monosaccharide branch and are also capable of hydrolyzing arabinan in pectic structures e.g. found in oil seeds such as rapeseed, sunflower and soybean. The linear arabinan is largely an α-1,5-homopolymer of arabinofuranose. Arabinofuranosidases closely related to *T*. *reesei* GH54 readily release arabinose from arabinan [56]. The LM6 antibody is specific for α-1,5-arabinan [57] and thus labelled the α-1,5-arabinan branches attached to pectin (rhamnogalacturonan I) present in cotyledon cell walls of rapeseed (Fig 7C) and sunflower seeds (Fig 8C). These signals faded away after incubation with MultiGrain (Figs 7D and 8D), indicating that most of the recognized antigen was degraded. Hence, the arabinofuranosidase found in the carbohydrase product was capable of reducing the arabinose branches of pectin to a high degree [58], but possibly left behind stumps of arabinan branches on the pectic backbone. The activity of the arabinofuranosidase was also confirmed by the results of the *in vitro* incubations of the rapeseed and sunflower samples (Table 3). The acid hydrolyzed solubilized fraction of the samples treated with MultiGrain showed more arabinose than control samples. The activity of the arabinofuranosidase on arabinogalactanans present in cereal cell walls could also explain the galactose measured in the acid hydrolyzed solubilized fraction of the cereal samples treated with MultiGrain.

Xyloglucan is the main hemicellulosic polysaccharide component of primary cell wall in dicotyledonous plant seeds, while mixed linked β-1,3(4)-glucanase and arabinoxylans are the predominant hemicelluloses in cereal grains [59, 60]. Immunocytochemistry visualization confirmed abundant presence of xyloglucan in DORB (Fig 6A), rapeseed (Fig 7A) sunflower (Fig 8A) and cassava (Fig 9A). Xyloglucans have been reported to be important component of cell wall structure in soybean, rapeseed, sunflower, cassava and other legumes commonly used as feedstuff [14, 61, 62]. Xyloglucans have α-1,4–linked glucosyl backbone that is heavily substituted by α-(1,6)-D-xylosyl residues. Generally, one in every four glucose units is left unsubstituted forming repeating (XXXG)n. The complexity of secondary substitution with other saccharides such as galactose, xylose, arabinofuranose, galacturonic acid and fucose varies and depends on the plant genotype and environmental factors [63]. Therefore, efficient degradation of xyloglucan structures requires synergistic action of both main chain and side chain enzymes. Common fungal secretomes such as *T*. *reesei*, *Aspergillus spp*. *Penicillium spp* have such combination of activities, but with some major differences. While the secretome from *A*. *niger* and *P*. *oxalicum* have more arabinofuranosidases as debranching enzyme, those from *T*. *reesei* have higher level of esterases for the same function [9] (Fig 1). Moreover, *T*. *reesei* and *A*. *niger* secretomes have one xyloglucan-specific β-1,4-endoglucanase belonging to the GH74 and GH12 families, respectively (Fig 1), while *P*. *oxalicum* secretome has no specific xyloglucan backbone degrading enzymes [9]. It is noteworthy to mention that many xyloglucan hydrolases have minor endo-1,4-β-D-glucanase (EG) activity, and many EGs have minor xyloglucan-hydrolyzing activity. However, the xyloglucanase (Cel74A) from *T*. *reesei* secretome is an exception as it acts on substituted motif on xyloglucan backbone [64, 65] and therefore has no activity on carboxymethylcellulose (CMC) and barley β-glucan [26]. This is possible because the Cel74A enzyme has a groove shape in the active site that allows for accommodation of the bulky side chains of xyloglucan polysaccharide [64, 66]. The direct chain-cutting Cel74A from *T*. *Reesei* is an endo-processive enzyme with regio-specificity on substituted backbone motifs. The enzyme acts initially on the internal bond of the substrate and then acts on the same substrate as an exo-enzyme, cleaving disaccharide residues. Based on the observations in the current study and regardless of the substrate sources, the final degradation of xyloglucan by Cel74A was very extensive (Figs 6B, 7B and 8B and Table 3). Such degradation showed to produce oligosaccharides and disaccharides [65] with potential prebiotic functionality, while dismantling the cell wall structure and decreasing viscosity (Table 2). Supplementation of MultiGrain to diets with sunflower meal (SFM) resulted in better performance in broiler chickens [67]. In the study, digestibility of the hemicellulose fraction of diets with 8 and 16% SFM was markedly enhanced (37 vs 54% and 15 vs 45%, respectively) by supplementation of the multicomponent carbohydrase product from *T*. *reesei*. In a different study a similar enzyme treatment resulted in improvement of weight gain and feed efficiency in broiler chickens fed diets with 10 and 20% rice bran inclusion [68].

Galactomannan polymers (galactose branched β-mannan backbone) are only found in low levels in common monogastric diets. Soybean meal (SBM) from dehulled soy contain a low level of galactomannan but consistently above 1% (1.26±0.14 n = 14) where the average galactose branching is 50% [69]. Less common raw materials such as palm kernel meal and copra meal have significantly higher galactomannan content with lower branching and hence solubility. The *T*. *reesei* secretome contained two enzymes characterized as mannanases (Fig 1). The first, Cel45A, has activity towards cellulose as well as high activity towards glucomannan (unbranched konjac glucomannan with 40% glucose) suggesting the enzyme cleaves at β-1,4-glucose residues [34]. Among the original *T*. *reesei* classified endoglucanases, Cel45A was the one with highest activity on glucomannan. The second mannanase, Man5A, selectively cleaves at the β-1,4-mannose residues, which allows for degradation of galactomannan having mannan backbone as shown in work conducted with locust bean gum [35, 70]. In this case the products were manno-oligosaccharides with galactose branches. The significant reduction in galactomannan viscosity shown could be directly linked to this mannanase (Man5A). The galactose branches in galactomannan are targeted by certain GH27 enzymes. The *T*. *reesei* alpha-galactosidase (GH27 Agl1) was shown to contribute to galactomannan degradation by removing α-1,6 linked galactose branches [36]. The three endoglucanases (Cel5A, Cel7B, Cel12A) have all also been reported to have some activity on glucomannan where they hydrolyze the glucosyl β-1,4 linkage, however to a lesser extent than the mannanases Cel45A and Man5A [34].

## Conclusion

The data presented in this paper characterised the multicomponent carbohydrase system of a *T*. *reesei* secretome and demonstrated its strong cell wall degradation capacity as measured by viscosity reduction, and polysaccharide degradation through *in vitro* incubations and fluorescence and immunocytochemistry confocal microscopy visualization. It appears that the natural balance of carbohydrate-degrading enzymes found in the *T*. *reesei* secretome can hydrolyze the diverse and complex cell wall structures in monocot and dicot grains or grain by-products commonly used in the animal feed industry.

## Supporting information

S1 Fig Confocal images of cross sections of whole wheat grains labelled with the immunofluorescent probe LM28 detecting glucuronoarabinoxylans with high degree of branching. The green colour indicates sample autofluorescence. Scale bar = 100 μm.
(TIF)

S2 Fig Confocal images of cross sections of whole barley grains labelled with the immunofluorescent probe LM28 detecting glucuronoarabinoxylans with high degree of branching. The green colour indicates sample autofluorescence. Scale bar = 100 μm.
(TIF)

S3 Fig Confocal images of cross sections of whole oat grains. (A) Section of oat incubated with sodium acetate buffer (control) at pH 5 for 3 h and subsequently stained with Calcoflour White. (B) Similar sections of oat incubated with the carbohydrase product for 3 hours and stained with Calcofluor White. Scale bar = 50 μm.
(TIF)

S4 Fig Confocal images of cross sections of whole oat grains labelled with the immunofluorescent probe detecting β-glucans. (A) oat section incubated with sodium acetate buffer (control) at pH 5 for 3 h and subsequently incubated with the antibody detecting the β-glucans structures. (B) similar section of oat incubated with the carbohydrase product for 3 hours and then with the antibody detecting β-glucans and the secondary red-fluorescence labelled antibodies. Red colour indicates binding of the antibody to the β-glucans and the green colour indicates sample autofluorescence. Scale bar = 50 μm.
(TIF)

S5 Fig Confocal images of cross sections of DORB labelled with the immunofluorescent probe LM11 detecting arabinoxylans with low degree of branching. The green colour indicates sample autofluorescence. Scale bar = 100 μm.
(TIF)

S6 Fig Confocal images of cross sections of whole rapeseed seeds labelled with the immunofluorescent probe LM25 detecting xyloglucans. The red colour indicates binding of the antibody to the xyloglucans and the green colour indicates sample autofluorescence. Scale bar = 100 μm.
(TIF)

S7 Fig Confocal images of cross sections of whole rapeseed seeds labelled with the immunofluorescent probe LM6 detecting (1–5)-a-L-arabinans. The red colour indicates binding of the antibody to the arabinans and the green colour indicates sample autofluorescence. Scale bar = 50 μm.
(TIF)

S8 Fig Confocal images of cross sections of whole cassava roots labelled with the immunofluorescent probe LM6 detecting (1–5)-a-L-arabinans. The red colour indicates binding of the antibody to the arabinans and the green colour indicates sample autofluorescence. Scale bar = 50 μm.
(TIF)
