# Supplementary figures and images for: Multicomponent carbohydrase system from Trichoderma reesei: A toolbox to address complexity of cell walls of plant substrates in animal feed
Source: PLoS One. 2021 Jun 4;16(6):e0251556. doi: 10.1371/journal.pone.0251556 (PMC8177525; doi:10.1371/journal.pone.0251556)

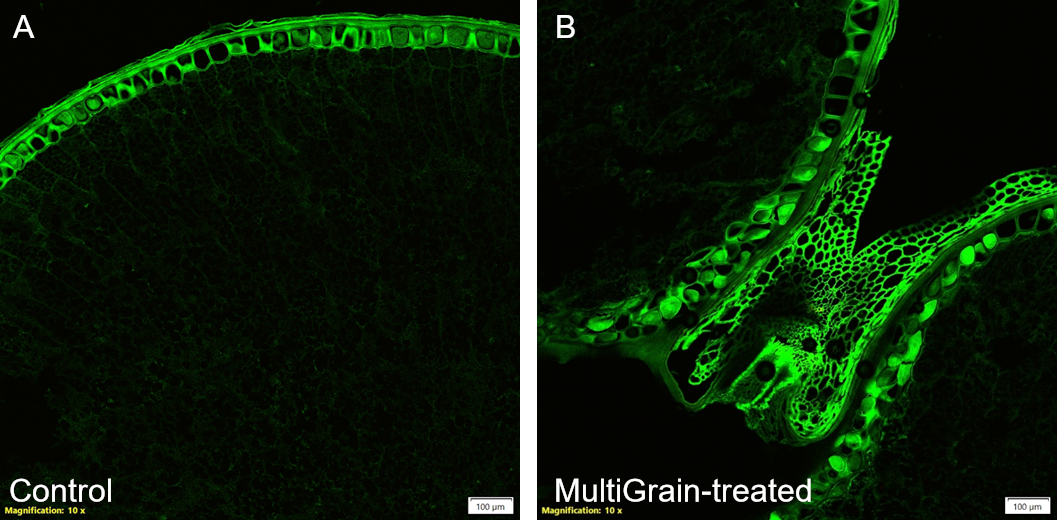

Supplement: S1 Fig — The green colour indicates sample autofluorescence. Scale bar = 100 μm. (TIF) [file pone.0251556.s001.tif]

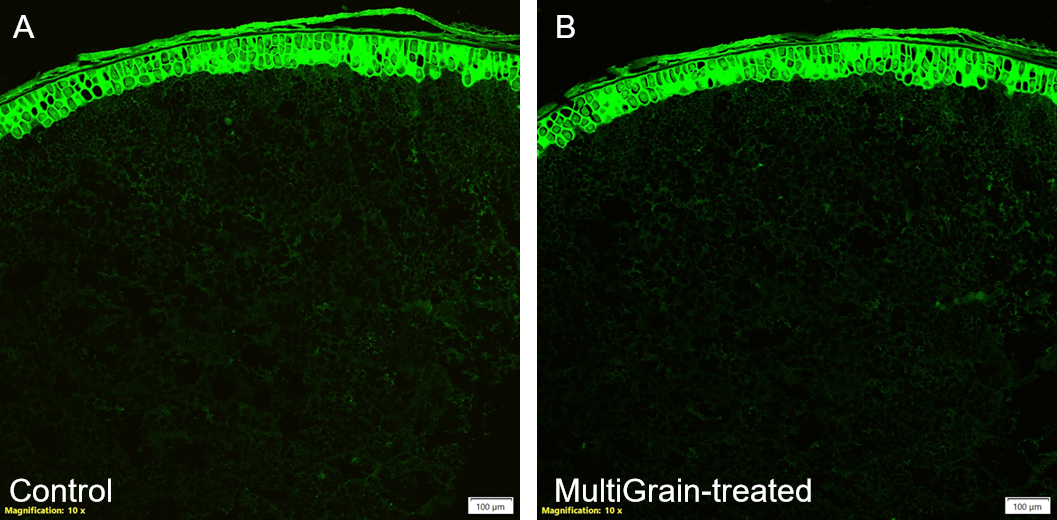

Supplement: S2 Fig — The green colour indicates sample autofluorescence. Scale bar = 100 μm. (TIF) [file pone.0251556.s002.tif]

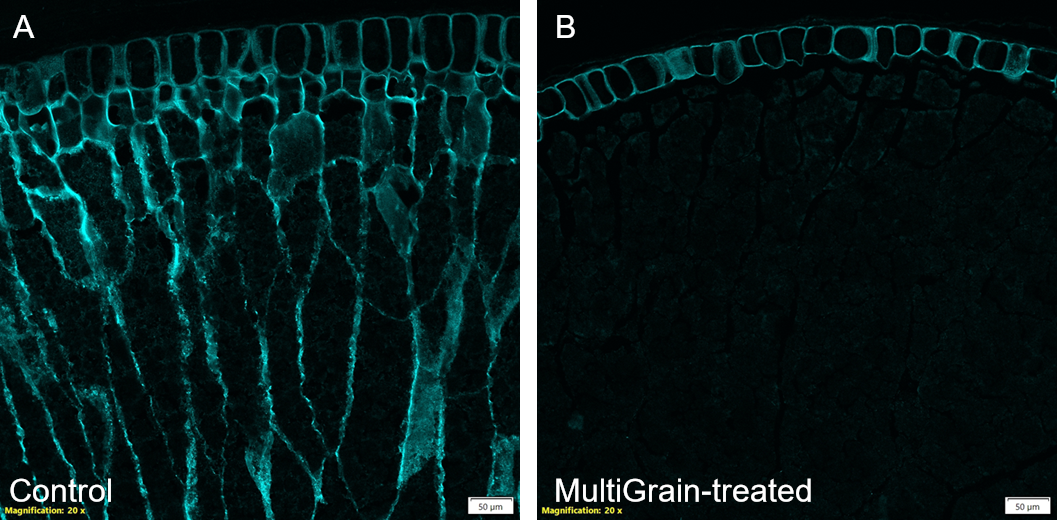

Supplement: S3 Fig — (A) Section of oat incubated with sodium acetate buffer (control) at pH 5 for 3 h and subsequently stained with Calcoflour White. (B) Similar sections of oat incubated with the carbohydrase product for 3 hours and stained with Calcofluor White. Scale bar = 50 μm. (TIF) [file pone.0251556.s003.tif]

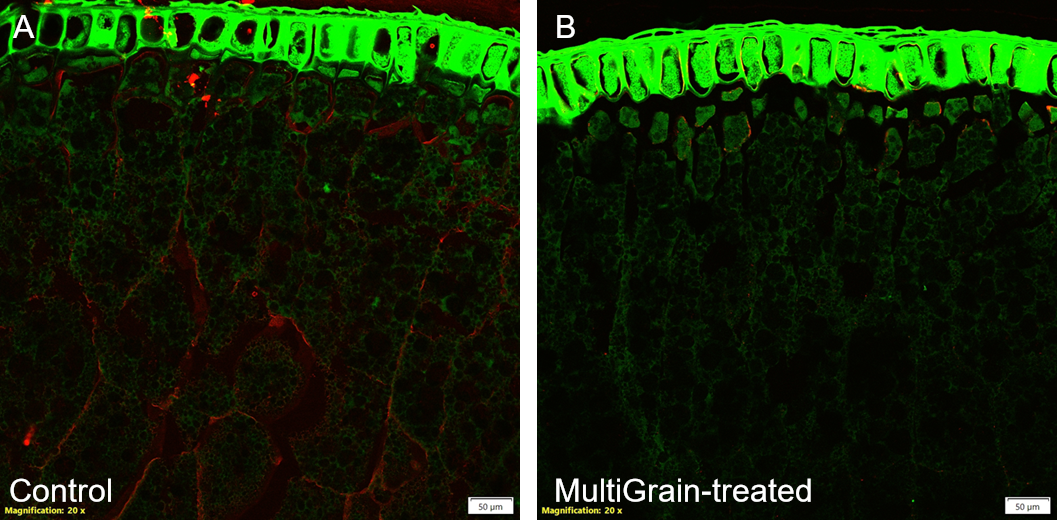

Supplement: S4 Fig — (A) oat section incubated with sodium acetate buffer (control) at pH 5 for 3 h and subsequently incubated with the antibody detecting the β-glucans structures. (B) similar section of oat incubated with the carbohydrase product for 3 hours and then with the antibody detecting β-glucans and the secondary red-fluorescence labelled antibodies. Red colour indicates binding of the antibody to the β-glucans and the green colour indicates sample autofluorescence. Scale bar = 50 μm. (TIF) [file pone.0251556.s004.tif]

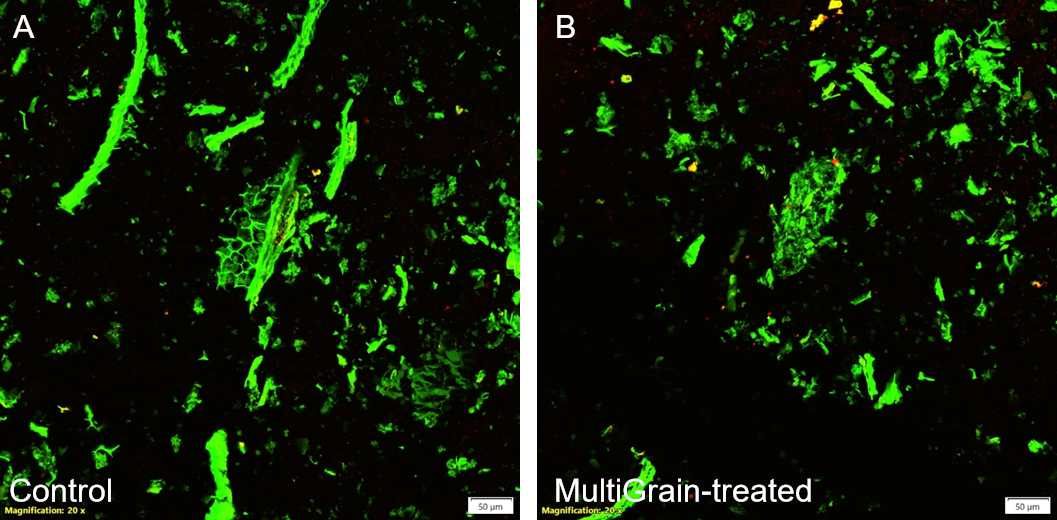

Supplement: S5 Fig — Scale bar = 100 μm. (TIF) [file pone.0251556.s005.tif]

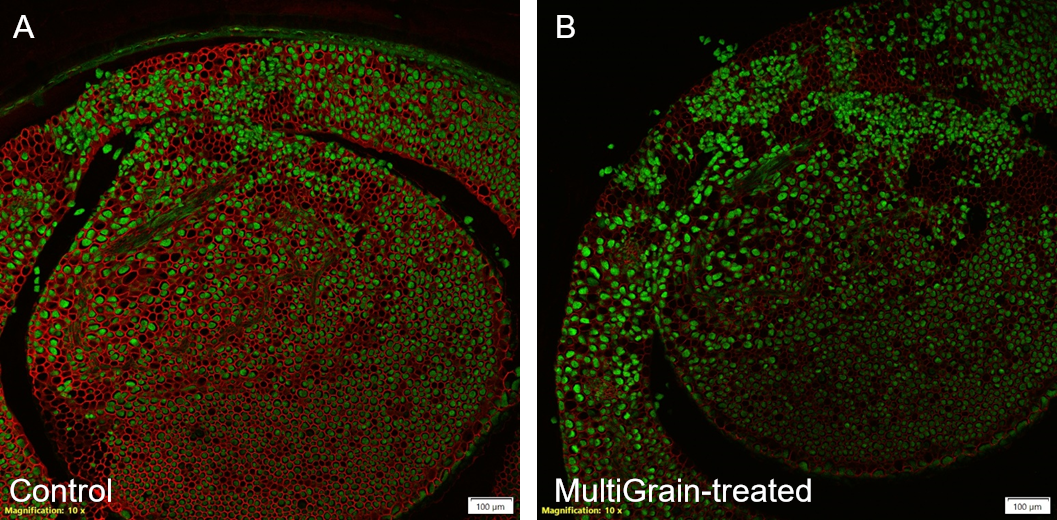

Supplement: S6 Fig — The red colour indicates binding of the antibody to the xyloglucans and the green colour indicates sample autofluorescence. Scale bar = 100 μm. (TIF) [file pone.0251556.s006.tif]

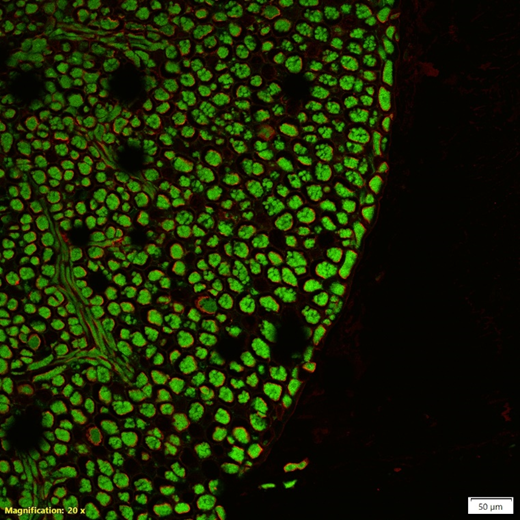

Supplement: S7 Fig — The red colour indicates binding of the antibody to the arabinans and the green colour indicates sample autofluorescence. Scale bar = 50 μm. (TIF) [file pone.0251556.s007.tif]

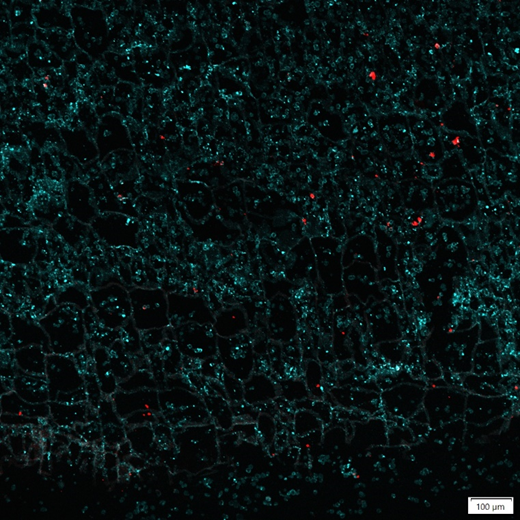

Supplement: S8 Fig — The red colour indicates binding of the antibody to the arabinans and the green colour indicates sample autofluorescence. Scale bar = 50 μm. (TIF) [file pone.0251556.s008.tif]
